# Metropolitan and Provincial Medical Schools: Their Cost and Course

**Published:** 1892-09-24

**Authors:** 


### Metropolitan and Provincial Medical Schools: their Cost and Course.

By a Provincial Physician.

Parents or guardians of those who wish to enter the medical profession very generally have some little difficulty in satisfying themselves upon many questions which naturally arise in regard to the cost of education, and the best way to attain the end in view—the winning of a legal qualification to practise the healing art. There are at least twenty corporate bodies in the United Kingdom which have the power of granting such qualifications after examination; and the number of separate qualifications open to competition is at least three times as great; but they are by no means all of equal merit and status. The standard of medical knowledge required of the candidates differs very greatly in the various examinations; and therefore the question, "How can a qualification be most easily obtained? is by no means equivalent to the inquiry, "How can I best qualify as a medical practitioner?"

It is very desirable that before entry the intending student should have some idea as to the kind of practice he hopes ultimately to attain to. If he aims at a high-class consulting practice, it is almost a *sine quâ non* that he should obtain the honourable, but honorary, position of physician or surgeon to some large hospital in a populous district. But to secure such appointments he will require a good University degree or one of the higher diplomas of one of the Royal Colleges of Physicians or Surgeons. Nor is this alone sufficient, for consultant work rarely comes to even the ablest men until they are well on in years; and, therefore, a certain amount of independent income is needed to tide over the waiting time. By a recent decision of the General Medical Council, the duration of medical education is five years at least. At the commencement of this period every student must be "Registered," and before this can be effected he must pass some "Preliminary" examination in subjects of general education, of which the following are compulsory: (1) English language, grammar, and composition; (2) Latin, including grammar and translation from specified authors, and of easy passages from other authors; (3) Mathematics, comprising arithmetic, algebra, to simple equations inclusive, and Geometry, which includes the subject matter of Euclid, Books I., II., and III., with easy deductions. One of the following optional subjects must be taken up: Greek, French, German, Italian, any other modern language, Logic. In future, the whole of the subjects must be passed in at the same time. The fee for the "Preliminary" is £1 1s. or £2 2s.

The cost of the purely professional education may be placed under two heads—(1) Fees payable for lectures and hospital practice, and (2) Fees payable to the examining bodies. The former vary considerably at the different medical schools in London; for example, the "Composition" fee to be paid in one sum at entry is at St. Bartholomew's Hospital £157 10s., and at Westminster £115. If paid by instalments the total is rather larger. The average at the provincial schools is decidedly less, and at none of them does the composition fee exceed £116. There is an impression abroad, which in certain quarters has been unfairly encouraged rather than corrected, that in the provincial schools the education is less thorough and complete than in London. Upon this point it should be sufficient to say that the practical results, based upon the reports of the various examining bodies, prove that the provincial schools have no reason whatever to fear comparison with their metropolitan competitors. Their lower fees will, doubtless, not be considered a disadvantage.

Then, again, all the large provincial hospitals draw their material for clinical instruction from a very wide area, both urban and rural, and from amongst a large and varied population. In this they cannot be said to be inferior to individual metropolitan institutions; and, indeed, the fact that there are so many smaller institutions in London (not generally available for the instruction of the ordinary medical student) for the treatment of special kinds of disease, and which divert the flow of such cases from the larger hospitals, tends, if anything, to prove that there must be a greater variety of cases for study in the large provincial hospitals than elsewhere. Another point worthy of consideration is, that in most of the metropolitan hospitals the number of students is greater in proportion to the number of patients in the wards than in the provincial hospitals; while in some they are actually more numerous than the patients; so that there must necessarily be far fewer opportunities for clinical investigation and instruction to individual students. By reason of the large numbers, too, it is hardly possible that individual students can receive so much attention and supervision from their teachers as is desirable.

For those who do intend to study in London, and who cannot then reside with relatives or real friends, the "residential colleges" which are attached to certain of the medical schools, offer a desirable home. Here the student is not left entirely to his own devices, but is subject to certain beneficial regulations and disciplinary guidance. There are such institutions in connection with St. Bartholomew's, Guy's, St. Mary's, King's College, and Middlesex hospitals.

If a student aims at obtaining University degrees he should be aware that the standard of the preliminary, as well as of the purely professional, examinations is comparatively high, though there are some few exceptions; and that, in consequence, the duration of his studies will almost certainly need to be extended beyond the legal minimum. With the exception of the University of London, which requires no residence, but admits to examination students from all parts of the empire, all the Universities expect the student to obtain the whole (or part) of his education within their own walls. This cosmopolitan character of the London University is a very great recommendation, and the high standard of medical knowledge required renders its degrees, to say the least, second to none in the whole world. The attempts recently made to institute an opposition University in the metropolis have ostensibly been purely for the benefit of those students who very naturally and reasonably desire an English University degree which shall be more easily obtainable than the existing London degrees; but the plan included a most objectionable proviso, that there should be a compulsory period of residence in London for all candidates from the provinces; thus exhibiting a most illiberal attempt to benefit the metropolitan colleges and medical schools at the expense of their provincial siscer institutions. Had the plan succeeded it would have been a grave scandal.

A few words now as to the examination expenses. These vary considerably at different places. The aggregate fees for the double qualification of Bachelor of Medicine and Bachelor of Surgery granted by the London University amount to only £20, and in this moderation a good example is set to other examining bodies who have the privilege of granting diplomas to practice. Thus the aggregate examination fees for the double qualification of "Licentiate of the Royal College of Physicians of London" and "Member of the Royal College of Surgeons of England" amount to no less than £36 15s. The corresponding cost in Scotland for the Royal Colleges' licence to practice is £26 5s., and in Ireland £34 13s. At the Scotch Universities the examination fees for the

Bachelor of Medicine and Master of Surgery amount to about £22, and at the Irish Universities £26.

The fee for the full medical and surgical qualification of the "Society of Apothecaries" of London is £15 15s. This society certainly deserves well of the medical profession in that it does try to safeguard the interests of those in practice, by enforcing the penalties of the law against "quacks" and other illegal practitioners. This is more than can be said for certain other corporate bodies who appear to think that after the reception of candidates' fees, the granting of diplomas to practice terminates their obligations to the profession at large.

When the student has passed a suitable preliminary examination he should communicate with the dean, secretary, or warden of the medical school he intends to join, asking for special information relative to "entrance scholarships," and other prizes. There are many more of these to compete for than was the case in former years; and some are so valuable as to provide a means whereby a hard-working, steady student may materially lessen, and in some cases even entirely defray, the cost of his education.

Entry as a student, followed by "registration," may be either in October, at the commencement of the six months' winter session, or in May, when the shorter summer session begins; preferably the former, for then the work of successive years falls more conveniently into place; and the time qualifications for most of the prizes are more favourable to the student.

